# American International Congress on Tuberculosis

**Published:** 1906-04

**Authors:** 


### American International Congress on Tuberculosis.

The officers of this Congress announce its next session for November 14, 15, and 16, 1906, at the city of New York.

The published call is as follows:

Office of the President,
Austin, Texas, February, 1906.

The executive officers of the American Congress on Tuberculosis, with the approval of its Governing Council, announce that the body will hold a Congress in the City of New York, commencing the 14th day of November, 1906, to be held three days.

All the officers, members and delegates are invited to attend, and to contribute papers to be read at the Congress, and to send the title of papers to the Secretary as early as possible.

This Congress will be open to members of all the professions, legislators, statesmen, the intelligent laity and the Rev. Clergy.

An enrolling fee of $3 is solicited to defray the expenses of a Bulletin, which should be sent forthwith to the Treasurer.

All the governments in the western hemisphere are invited to send delegates to this Congress and to co-operate in its labors.

The public press, lay and medical, are hereby invited to give publicity to this announcement.

F. E. Daniel, M. D., President,
Austin, Texas.
Matthew M. Smith, M. D., Secretary,
Austin, Texas.
Clark Bell, Esq., LL. D., Treasurer,
39 Broadway, New York.

The foregoing announcement and call for the session of the American International Congress on Tuberculosis as signed by the executive officers, is hereby approved by a majority of the Governing Council and Board of Executive Officers.

MEMBERS OF THE COUNCIL.

Dr. J. Mount Bleyer, of New York City.
Dr. A. K. Foster, State Board of Health, Sacramento, Cal.
Dr. A. P. Grinnell, 500 Fifth Avenue, New York City.
Dr. H. Edwin Lewis,, New York City.
Dr. Andrew C. Smith, President State Board of Health, Portland, Oregon.

EX-MEMBERS OF COUNCIL.

Moritz Ellinger, New York City.
M. K. Kassabian, M. D., Philadelphia, Pa.
R. J. Nunn, M. D., Savannah, Ga.
W. F. Drewry, M. D., Petersburg, Va.
J. W. P. Smithwick, La Grange, N. C.

This call is intended to be addressed to every member of the

American International Congress on Tuberculosis, and to each and every delegate accredited to the Congress since its organization, in February, 1900, and to all citizens interested in the conflict for the suppression of tuberculosis. It will be sent especially to all boards of health and organizations against tuberculosis in the western hemisphere, including State, provincial, and county medical organizations, who it is hoped will send delegates from each body and advise us of name and address.

The Governors of the various States whose names are now on its lists of vice-presidents are requested to give this notice publicity, both in the States, Territories and dependencies of the American Union, and also the executives of all the governments in the western hemisphere, with the Governors of the States and Provinces, are requested to give this announcement publicity, so that it may reach men of science in all those countries interested in the conflict with tuberculosis, so that they may co-operate in the great problems of preventive legislation against tuberculosis therein; municipal and governmental sanitariums; and all the questions involved in the struggle, with the great scourge of the race.

The Bulletin of the Congress of 1904 is now ready and has been sent to subscribers. So long as the edition lasts it will be sent on application to the *Medico-Legal Journal* on receipt of $2. It is a large volume of 476 pages, with a large number of illustrations. If any subscriber has not received the Bulletin he is requested to apply for it at once.

The *Medico Legal Journal* will send a copy of Vols. 22, 23, and 24 to any member or delegate to this Congress at half price, $1.50, if paid in advance.

Members and delegates who desire to enroll will remit the enrolling fee of $3, and will be entitled to receive the Bulletin of the Congress free, if enrolling fee is sent to the Treasurer, Clark Bell, 39 Broadway, New York.

Members of the organization who do not wish to enroll can retain their place as members on payment of the annual dues of $1.

Contributions to defray the expenses of the Congress are solicited by the executive officers.

HONORARY PRESIDENTS.

*Lay.*

Hon. General Russell A. Alger, ex-Secretary of War, U. S. Senator from Michigan, Detroit, Mich.

Hon. Clark Bell, LL. D., Chairman Committee on Organization; Chairman Executive Board and President of the Medico-Legal Society, New York City.

Hon. Porfirio Diaz, President of Republic of Mexico, City of Mexico.

Hon. Moritz Ellinger, Esq., President of the Council; ex-Coroner of New York City; Corresponding Secretary and Honorary Member of the Medico-Legal Society, New York City.

Hon. L. A. Emery, Justice Supreme Court of Maine; Honorary Member Medico-Legal Society; Professor Medical Jurisprudence, Bowdoin College, Ellery, Maine.

Hon. W. S. Fielding, Minister of Finance, Dominion of Canada, Ottawa, Canada.

Hon. Charles G. Garrison, Justice of the Supreme Court of New Jersey; Honorary Member Medico-Legal Society, Camden, N. J.

*Hon John Hay, American Secretary of State; Honorary Member Medico-Legal Society, Washington, D. C.

Hon. Stephen B. Elkins, U. S. Senator from West Virginia, Washington, D. C.

Hon. James Loudon, President of the University of Toronto; Honorary Vice-President of the Congress, Toronto, Ontario.

Hon. Senor Ignacio Mariscal, Minister of Foreign Relations of Mexico, City of Mexico.

*Medical.*

Dr. Wm. Bayard, Hon. Vice-President for New Brunswick, St. John, N. B.

Dr. A. N. Bell, Editor *Sanitarian;* ex-President American Congress on Tuberculosis; Honorary Member Medico-Legal Society, Brooklyn, N. Y.

Dr. E. J. Barrick, M. D., M. R. C. S., Eng.; L. R. C. P. and S., London and Edinburgh; ex-President American Congress on Tuberculosis, Toronto, Ont.

W. B. Fletcher, M. D., late Superintendent State Hospital for Insane; Vice-President American Congress on Tuberculosis, Indianapolis, Ind.

Hon. L. F. C. Garvin, M. D., Honorary Vice-President of the Congress; Governor of Rhode Island.

Sir William Hingston, M. D., M. P., Honorary Vice-President of the American Congress on Tuberculosis, Montreal, Canada.

Prof. Dr. Charles H. Hughes, Editor of the *Alienist and Neurologist;* Honorary Member of the Medico-Legal Society, St. Louis, Mo.

Prof. Dr. Herman Kornfeld, Honorary Vice-President of the Congress; Honorary Member of the Medico-Legal Society, Gleiwitz, Germany.

Hon. Miguel A. Otero, M. D., Honorary Vice-President of the Congress; Governor of New Mexico, Santa Fe, N. M.

Hon. Geo. C. Pardee, M. D., Governor of California; Honorary Vice-President of the Congress; Secretary State Board of Health, Sacramento, Cal.

General Presley M. Rixie, M. D., Surgeon General U. S. Army, Washington, D. C.

General Nicolas Senn, M. D., Surgeon-General of Illinois, Chicago, Ill.

Prof. Dr. Otto Von Schroen, Royal University of Naples, Naples, Italy.

